# The Role of Chromatin Modifications in the Evolution of Giant Plant Genomes

**DOI:** 10.3390/plants12112159

**Published:** 2023-05-30

**Authors:** Andrew R. Leitch, Lu Ma, Steven Dodsworth, Jörg Fuchs, Andreas Houben, Ilia J. Leitch

**Affiliations:** 1 School of Biological and Behavioural Sciences, Queen Mary University of London, London E1 4NS, UK; lu.ma@usc.edu.cn (L.M.); steven.dodsworth@port.ac.uk (S.D.); 2 Royal Botanic Gardens, Kew, Richmond, Surrey TW9 3AB, UK; i.leitch@kew.org; 3 School of Biological Sciences, University of Portsmouth, Portsmouth PO1 2DY, UK; 4 Leibniz Institute of Plant Genetics and Crop Plant Research (IPK), Gatersleben, 06466 Seeland, Germany; fuchs@ipk-gatersleben.de (J.F.);

**Keywords:** histone modifications, giant genomes, chromatin, immunocytochemistry, dark matter, epigenetics

## Abstract

Angiosperm genome sizes (GS) range ~2400-fold and comprise genes and their regulatory regions, repeats, semi-degraded repeats, and ‘dark matter’. The latter represents repeats so degraded that they can no longer be recognised as repetitive. In exploring whether the histone modifications associated with chromatin packaging of these contrasting genomic components are conserved across the diversity of GS in angiosperms, we compared immunocytochemistry data for two species whose GS differ ~286-fold. We compared published data for *Arabidopsis thaliana* with a small genome (GS = 157 Mbp/1C) with newly generated data from *Fritillaria imperialis*, which has a giant genome (GS = 45,000 Mbp/1C). We compared the distributions of the following histone marks: H3K4me1, H3K4me2, H3K9me1, H3K9me2, H3K9me3, H3K27me1, H3K27me2, and H3K27me3. Assuming these histone marks are associated with the same genomic features across all species, irrespective of GS, our comparative analysis enables us to suggest that while H3K4me1 and H3K4me2 methylation identifies genic DNA, H3K9me3 and H3K27me3 marks are associated with ‘dark matter’, H3K9me1 and H3K27me1 mark highly homogeneous repeats, and H3K9me2 and H3K27me2 mark semi-degraded repeats. The results have implications for our understanding of epigenetic profiles, chromatin packaging and the divergence of genomes, and highlight contrasting organizations of the chromatin within the nucleus depending on GS itself.

## 1. Introduction

Angiosperm genome sizes (GS, reported here as 1C-values, i.e., the amount of DNA in the unreplicated gametophytic nucleus) range ~2400 fold, from ~61 Mbp/1C in *Genlisea tuberosa* to 148,852 Mbp/1C in *Paris japonica* [1]. Such an enormous range in GS provides opportunities to study whether GS itself plays a role in influencing DNA evolution and how the DNA is packaged within the nucleus, i.e., chromatin structure. Nevertheless, most research on GS and genome structures in angiosperms has focused on model plant species with small genomes, e.g., *Arabidopsis thaliana* (157 Mbp/1C), which is nearly 1000 times smaller than the largest plant genomes known. At the diploid level, the largest known genomes are found in the genus *Fritillaria* (Liliaceae, reaching ~100,000 Mbp/1C; pers. comm.) and potentially also in *Viscum album* (Viscaceae, ~100,000 Mbp/1C [2], although no chromosome count was made to confirm the ploidy of the individual analysed).

The size of angiosperm genomes reflects their evolutionary history of polyploid events as well as rates of repeat accumulation via, for example, recombination and (retro)transposition, and the number and frequency of DNA insertion/deletion events [3,4]. Excluding polyploidy, the key processes that are thought to be important in influencing the accumulation and deletion of repeats, and hence GS, are (retro)transposition, unequal recombination (especially impacting tandem repeats), and DNA repair mechanisms, which are reported to act differently in large and small genomes (e.g., [5,6,7]). All of these processes are also influenced by the activity of RNA-directed DNA methylation (RdDM), maintenance methylation pathways, and the types of histone modifications. Potentially, any failure of these epigenetic processes could lead to uncontrolled amplification of repeats, resulting in species with large GS. In contrast, Fedoroff [8] suggested that the evolution of large genomes arose as a result of an overly efficient regulation of repeats via epigenetic-driven heterochromatin formation, stating “I contend that it was precisely the evolution of prokaryotic mechanisms to regulate homologous recombination within the eukaryotic genome that made it possible for the genomes to grow”.

Recent studies have shown that the genomes of *Fritillaria* [9], and indeed all angiosperm species studied with similarly large GS, have repeat abundances that fall far short of expectation [10]. In these genomes, large quantities of low or single-copy non-genic sequences, perhaps accounting for nearly half of the genome, were observed. Such DNA is considered to mainly comprise the so-called ‘dark matter’ of the genome, i.e., DNA that is not genic, not associated with genes or their regulation, or highly repetitive [10,11]. The repeat content of these large genomes is also more heterogenous than would be expected, given what we know from the repeat content of species with smaller GS [10]. The data lead to the hypothesis that repetitive DNA dynamics, comprising insertions and excisions, change in species with a GS above about ~10,000 Mbp/1C. In species with smaller GS than this threshold, repeats are typically amplified and deleted rapidly, leading to a repeat half-life of a few million years and a repeat content that is relatively homogeneous. In these species, the proportion of the genome that is repetitive is thought to increase with increasing GS, reaching up to ~90% of the genome in species with GS around 10,000 Mbp/1C [10,12,13]. In contrast, for species with GS greater than ~10,000 Mbp/1C, the frequency of repeat amplification exceeds excision, leading to genome enlargement. Given their slow removal, this results in the accumulation of repeats that slowly mutate and degrade over time to a point where they are no longer recognisable as repeats and instead give rise to the ‘dark matter’ [9,10].

To better understand genome dynamics in species with large GS, previous studies questioned whether the epigenetic machinery that impacts DNA methylation, chromatin compaction, and hence repeat dynamics, might be different in species with large GS compared to what we know from studying species with small genomes (i.e., <~10,000 Mbp/1C). Consequently, the epigenetic machinery in *Fritillaria persica* (39,000 Mbp/1C) was examined to look for evidence of any unusual features in the epigenetic machinery involved in the RdDM pathway for repeat silencing. In that study, abundant 24 nucleotide small RNAs and cytosine methylation at CG and CNG motifs were observed, indicating that the RdDM pathway was active and functioning [14]. Furthermore, most but not all genes involved in the RdDM pathway were expressed in *F. persica*. While it is difficult to distinguish ‘missing’ from ‘not found’, the data nevertheless suggest some deficiency/differences in the activity of the RdDM pathway in this large genome species [15].

To extend the analysis of Ma et al. [15], here, we compare the global patterns of histone methylation in the large genome of *F. imperialis* (45,000 Mbp/1C; [9]) with published data from the much smaller genome of *A. thaliana*. Previously it has been shown that the methylation of lysine 4 in histone H3 (H3K4me) is a ubiquitous mark of open chromatin configuration (i.e., euchromatin) across eukaryotes, including plants [16,17]. However, comparative studies across 24 angiosperm species showed that the distribution pattern of other post-translational histone modifications appeared to be less conserved [16]. For example, dimethylation of lysine 27 on histone H3 (H3K27me2) was suggested to be a heterochromatic, chromocentre marker in *A. thaliana* and yet appeared to label euchromatin in barley (*Hordeum vulgare*, 5428 Mbp/1C) [17]. In addition, H3K9me1 was suggested to be a mark for heterochromatin in angiosperms, but the same mark appeared to mark euchromatin in the large genomes of the gymnosperms *Picea abies* (19,610 Mbp/1C) and *Pinus sylvestris* (22,520 Mbp/1C) [18]. Whilst such variability might represent species-specific or group-specific differences, they might also provide evidence that histone modification patterns may differ depending on the GS of a species. Potentially, the different distribution patterns reported for plant species may represent the different distributions and abundances of repetitive elements and their relationships with genic domains and/or ‘dark matter’. To build on previous studies, we compare the distribution of histone modifications between *F. imperialis* and *A. thaliana*, which differ ~286 times in GS.

## 2. Results

### 2.1. The Repeat Profile of Fritillaria imperialis Is Characterized by a Large Fraction of Ty3/Gypsy Retroelements

**Figure 1:**
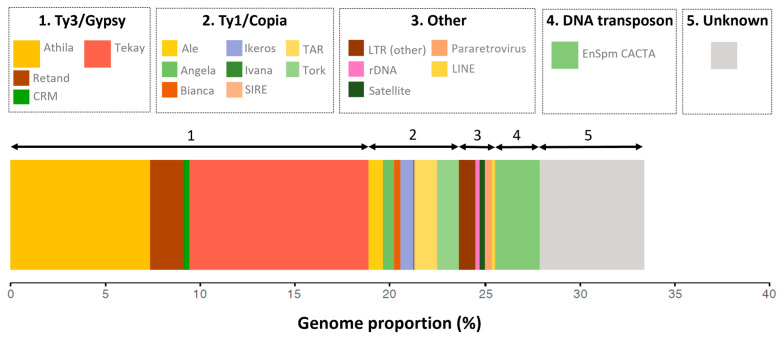
Profile of the repeat landscape in *Fritillaria imperialis* generated via RepeatExplorer2 [20]. Larger coloured boxes in the legend are shown for Athila (~7.4%) and Tekay (~9.5%) Ty3/Gypsy elements and the EnSpm CACTA-like DNA transposon (~2.3%). Unknown repeat types are shown in grey and amount to 5.5%. Repeat abundances less than 0.05% are not shown.

Genome skimming approaches, using the equivalent of 2.3% of the *F. imperialis* genome (10.5 million reads), revealed that repeats (i.e., any sequence present in more than ~100 copies per genome) represent about a third of the genome. The read numbers in clusters, with a genome proportion of 0.01% or greater, collectively accounted for about 33.4% of the genome (Table 1, Figure 1).

The most common single repeat type was a Ty3/Gypsy retroelement of the Tekay lineage of chromoviruses [19], of which one supercluster contributed ~7.2% of the genome, and together (i.e., all Tekay annotated clusters), these elements accounted for ~9.5% of the genome. The second most common repeat cluster was another Ty3/Gypsy retroelement of the Athila family (4.2%), and together with other Athila clusters, these elements comprised ~7.4% of the genome. The remaining repeats in the top 10 most abundant clusters consisted mostly of other Ty3/Gypsy and Ty1/Copia retroelements, with the notable exception of an EnSpm CACTA-like DNA transposon that accounted for ~2.3% of the genome. Though no one particular repeat accounted for a huge proportion of the genome, Ty1/Copia and Ty3/Gypsy elements together comprised most of the repeats identified (~24% of the genome). These data are consistent with our previous report of repeat composition in eight species of *Fritillaria*, including *F. imperialis*, which showed that repeats contributed a relatively low fraction of the genome and that the repeat content was characterised by a diversity of typically low abundant repeats [9]. The remaining ~66% of the genome comprised low copy (<100 copies) repeats, semi-degraded repeats, ‘dark matter’, genes, and their regulatory regions.

### 2.2. The Global Chromatin Landscape of Fritillaria imperialis Compared with Arabidopsis thaliana

**Figure 2:**
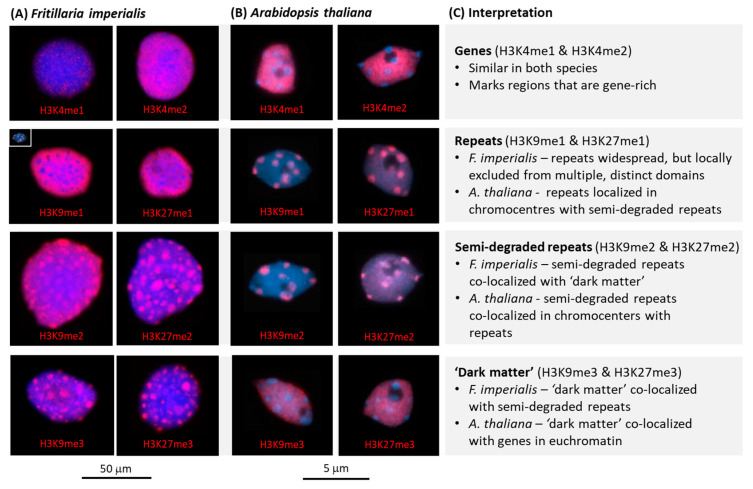
Similarities and differences in the distributions of specific methylated histone H3 marks of (**A**) *Fritillaria imperialis* (this study) and (**B**) *Arabidopsis thaliana* (images of labelled nuclei taken from Fuchs and Schubert, [21]), which differ ~286-fold in genome size, together with (**C**) the interpretation of the histone methylation targets. Immunolabelling with antibodies against specific methylation marks (me1, me2, and me3) on lysines K4, K9, and K27 of histone H3 are visible as pink fluorescent signal on interphase nuclei that were counterstained with DAPI (blue fluorescent signal). Scale bars are shown, but to better illustrate the differences in nuclear size between *A. thaliana* and *F. imperialis,* a nucleus of each species is shown at the same magnification in (**A**) labelled for H3K9me1.

We conducted immunostaining to interphase nuclei of *F. imperialis* using antibodies that discriminate between the number of methyl groups (i.e., me1, me2, and me3) on lysine K4, K9, and K27 of histone H3 (Figure 2).

#### 2.2.1. Labelling of *Arabidopsis* and *Fritillaria* Nuclei to Detect Histones H3K4me1 and H3K4me2

In *F. imperialis,* labelling with the antibodies against H3K4me1 and H3K4me2 gave rise to a low density of pink signals across the nucleus, although with H3K4me2, the signal was more widespread and intense than H3K4me1 (Figure 2A). The same antibodies are reported to label euchromatin in regions outside the chromocentres in *A. thaliana* and not to label the chromocentres themselves, which instead appear as bright blue, DAPI-positive, and label-free circular areas in interphase nuclei (Figure 2B, from reference [21]). It is possible that the different signal intensities between *A. thaliana* and *F. imperialis* nuclei are a consequence of differences in the quality and/or dilutions of antibodies used or associated with the different GS (see insert in Figure 2A: H3K9me1, which shows an *A. thaliana* nucleus at the same magnification as an *F. imperialis* nucleus, whose GS is ~286 times larger).

#### 2.2.2. Labelling of Histones H3K9me2, H3K9me3, H3K27me2, and H3K27me3

The labelling patterns observed with antibodies against H3K9me2, H3K9me3, H3K27me2, and H3K27me3 in *F. imperialis* appeared to be broadly similar to H3K9me1, H3K9me2, H3K27me1, and H3K27me2 for *A. thaliana*. However, it is noted that in *F. imperialis*, the spots of the pink signal were much larger than in *A. thaliana*, as each spot was similar in dimensions to an entire *A. thaliana* nucleus (see inset in Figure 2A).

#### 2.2.3. Labelling of Histones H3K9me1 and H3K27me1

In contrast to the labelling of H3K9me2, H3K9me3, H3K27me2, and H3K27me3 (as above), the nuclei of *F. imperialis* appeared to have substantially different distributions for H3K9me1 and H3K27me1, as the pink signal was widely dispersed across the nucleus, except in distinct ‘holes’ that were devoid of this epigenetic mark (visible as blue areas inside a pink background, Figure 2A). Indeed, the labelling of H3K9me1 and H3K27me1 appeared to be the mirror image of H3K9me2, H3K9me3, H3K27me2, and H3K27me3, with the unlabelled blue ‘holes’ seen for H3K9me1 and H3K27me1 being approximately the same size as the roughly circular pink labelled areas for H3K9me2, H3K9me3, H3K27me2, and H3K27me3. In contrast, for *A. thaliana* the antibodies against H3K9me1 and H3K27me1 appeared to preferentially label chromocenters (Figure 2B).

## 3. Discussion

### 3.1. Global Distribution of Post-Translational Histone Modification

The RepeatExplorer2 analysis to characterize the repetitive fraction of the genome suggests that the large *Fritillaria imperialis* genome comprises ~34% repeated DNA (Table 1). Given protein-coding genes and their associated regulatory regions (i.e., functional gene space) are estimated to make up <1% of the genome [9], the remaining ~66% of the genome is assumed to be largely composed of sequences that lack sufficient sequence homology to be recognised as repeats and hence are considered to be semi-degraded repeats and ‘dark matter’. Similar results for other species in the genus *Fritillaria* have been published previously [9,10].

Given the enormous size of the *F. imperialis* genome (45,000 Mbp/1C) in contrast to most angiosperm species (modal GS of angiosperms = 587 Mbp/1C, [1]), the question arises as to whether there are any differences in how the different components of the genome (i.e., functional gene space, repeats, semi-degraded repeats, and ‘dark matter’) are labelled by antibodies against various histone modifications. From our analyses of *F. imperialis* presented here and comparisons with comparable data from *A. thaliana* [21], it is clear that the immunolabelling results reveal both notable similarities and differences in the distribution patterns of histone 3 methylation variants between these species.

It is thought that H3K4me1 and H3K4me2 mark euchromatin and H3K9me1 and H3K27me1 mark heterochromatin [17], and the distribution of antibody labelling in the two focal species support this (Figure 2A,B, see below). However, there appear to be substantial differences in the distribution of the immunolabelling of H3K9me1 and H3K27me1 compared with H3K9me2, H3K27me2, H3K9me3, and H3K27me3 between the two species. One possible explanation for the differences in the distribution of H3K9me2 and H3K27me2 compared with H3K9me1 and H3K27me1 in *F. imperialis* and *A. thaliana* is that these different histone variants are targeting specific types of repeats, which differ in their abundance, distribution, and chromatin packaging across the genome between species. Hence, it is possible that in species with giant genomes such as *F. imperialis*, the repeat types targeted by H3K9me2 and H3K27me2 occur in spatially distinct domains from other repeat types targeted by H3K9me1 and H3K27me1. If so, this would give rise to contrasting labelling patterns compared to the small genome of *A. thaliana*, where the spatial resolution may be insufficient to detect such differences. However, such an explanation of the data does not account for the ~66% of the genome that is not highly repetitive in *F. imperialis*, i.e., the semi-degraded repeats and ‘dark matter’. In short, if repeats fully accounted for the distribution of these four histone marks, we would expect more than half the genome to be unlabelled by these antibodies, which is clearly not the case (Figure 2A).

To account for the semi-degraded repeats and ‘dark matter’ of the genome, and assuming antibodies label the same component of DNA/chromatin in all species irrespective of GS, we propose the following interpretations of the immunocytochemistry data presented for *F. imperialis* and the equivalent data previously published for *A. thaliana* and other species with GS up to ~22,000 Mbp/1C [21].

#### 3.1.1. H3K4 Methylation Marks Genes and Associated Regulatory Regions

Similar labelling patterns with antibodies against H3K4me1 and H3K4me2 have been reported in 24 angiosperm species that ranged in GS from 170 Mbp/1C to 7760Mbp/1C) [16]. It is likely that H3K4 methylation is a euchromatin-specific mark, which is highly conserved and marks genic domains [17] irrespective of GS. In *A. thaliana,* antibodies against H3K4me1 and H3K4me2 generated dispersed signals in euchromatic regions outside of chromocentres, while in *F. imperialis*, they generated weak, light speckling of signals across the nucleus (Figure 2A,B). If we assume that there are ~30,000 genes in *A. thaliana* and *F. imperialis*, each ~1000 bp in length, then this amounts to about 3 × 10^7^ bp (30 Mbp) of DNA. In *A. thaliana*, with a GS of 157 Mbp/1C, the genic domains, therefore, amount to ~20% of the genome, whilst in *F. imperialis* with a GS of 45,000 Mbp/1C, they comprise only a tiny fraction (~0.07%) of the genome.

The light speckling of signals observed across the giant genome of *F. imperialis* (Figure 2A) is similar to that reported across the large chromosomes of the gymnosperm *Pinus sylvestris* (GS 22,000 Mbp/1C, [18]), highlighting the highly conserved targeting of H3K4 methylation in euchromatin beyond angiosperms. Indeed, studies across a huge diversity of species now point to the universality of these H3K4 methylation variants labelling genic domains across eukaryotes [21].

#### 3.1.2. H3K9me3 and H3K27me3 Are Associated with the ‘Dark Matter’ of the Genome

‘Dark matter’ represents uncharacterised sequences that are non-genic and of unclear origin, although most are likely to comprise highly degraded repeats that are no longer identifiable as being repetitive and are essentially low/single copy sequences that no longer form part of the ‘repeatome’ [22]. In *A. thaliana*, antibodies against H3K9me3 and H3K27me3 show similar labelling patterns to those observed with antibodies against methylated H3K4 residues (Figure 2B). However, if H3K9me3 and H3K27me3 are marking the same types of sequences in both *A. thaliana* and *F. imperialis*, then it is unlikely they are marking genic domains as proposed for H3K4 methylation (see Section 3.1.1). This is because of the very different labelling pattern observed in *F. imperialis* compared to that for *A. thaliana*. In *F. imperialis*, we see that antibodies against H3K9me3 and H3K27me3 produce large pink spots, with each spot being similar in size to an entire *A. thaliana* nucleus (Figure 2A) despite the above estimates of the total genic content of the *F. imperialis* genome comprising only ~0.07% of the DNA. Similar localised signals were also reported on the large chromosomes of the gymnosperm *P. sylvestris* [18]. We, therefore, propose that the antibodies against H3K9me3 and H3K27me3 are labelling ‘dark matter’ given that the signal occurs outside of chromocentres in *A. thaliana* (Figure 2B). As noted above, we know that ‘dark matter’ forms a substantial component of the DNA in the *F. imperialis* genome, perhaps accounting for nearly 66% of the genome (see Section 2.1); hence, this is consistent with the size of the signals observed in *F. imperialis* with these antibodies (Figure 2A).

#### 3.1.3. H3K9me1 and H3K27me1 Associated with Repeats

It is probable that H3K9me1 and H3K27me1 modifications label repeats based on studies of plant species across the range of GS [17]. In *A. thaliana*, repeats account for approximately 32% of the genome [22], predominantly comprising retroelements (Ty1/Copia and Ty3/Gypsy elements), DNA transposons, and satellite repeats. These repeats, when labelled with H3K9me1 and H3K27me1 antibodies, highlight chromocentres—regions of heterochromatic DNA (Figure 2B). If this interpretation observed in *A. thaliana* is extended to *F. imperialis*, then repeats in heterochromatic DNA will also be labelled with H3K9me1 and H3K27me1. Indeed, in *F. imperialis*, we observed that the immunolabelling with these antibodies generated signals across the entire nucleus except for large, circular unlabelled areas (‘holes’), which we predict contain the ‘dark matter’ (see Section 3.1.2).

Overall, a model for the chromatin organization of the *F. imperialis* genome is emerging where repetitive DNAs occupy spatially distinct domains from ‘dark matter’. It is notable that the large genome of *Pinus sylvestris* also appears to be similarly organized, as seen from the similar labelling pattern observed with the H3K9me1 and H3K27me1 antibodies reported by Fuchs et al. [18]. Thus, the feature of a highly compartmentalised genome of repeats and ‘dark matter’ for such large genomes as *F. imperialis* is just as it is in a small genome, just in a nucleus that has 286 times more DNA.

#### 3.1.4. H3K9me2 and H3K27me2 Associated with Semi-Degraded Repeats

An analysis of the chromosomal distribution of H3K9me2 and H3K27me2 in *A. thaliana* revealed labelling at chromocentres, which suggests they are marks for heterochromatin. However, if these antibodies are labelling the same class of DNA across species, they are unlikely to be marks for repetitive DNA. This is because the same antibodies label large spots across the nucleus of *F. imperialis*, areas that we argue in Section 3.1.2 above are areas of ‘dark matter’. Instead, we speculate that these antibodies are labelling semi-degraded repeats, which in *A. thaliana* are found in the chromocenters together with repeats, while in *Fritillaria*, they are found in separate domains co-localized with the ‘dark matter’. However, even in *A. thaliana*, high-resolution analysis of centromeric domains shows that they comprise large blocks of tandem repeats that have a centromeric function, separated by flanking sequences of transposable elements that label with H3K9me2 [23]. It is likely that H3K9me2 and H3K27me2 are labelling semi-degraded transposable elements in these flanking domains. Our interpretation is supported by a recent analysis of DNA sequences in *A. thaliana*, which suggested that partially, semi-degraded yet still identifiable repeat sequences together accounted for about 50% of the genome [24] and were predominantly but not entirely found associated with the repeat component of the genome [22].

#### 3.1.5. Predictions for Future Proposed Research

**Figure 3:**
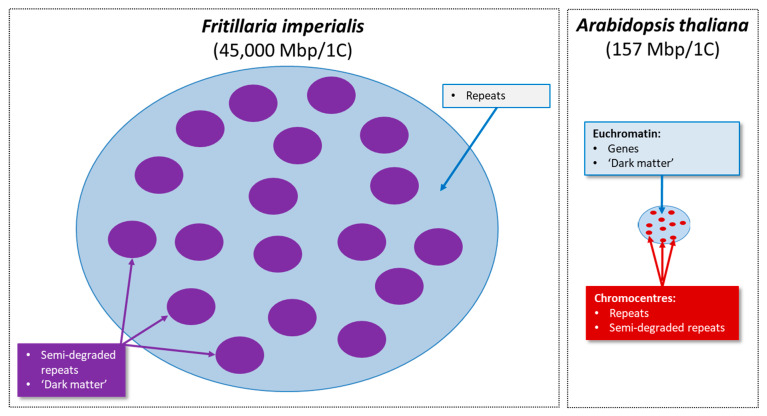
Diagrammatic summary of the distribution of different genomic domains in *Fritillaria imperialis* and *Arabidopsis thaliana* differing by ~286-fold in genome size. Genes in *F. imperialis* appear to be widely dispersed but comprise only a tiny fraction of the genome (i.e., ~0.07%), and it is unknown if they are localised to any particular domain. Note that the purple ‘spots’ in *F. imperialis* predicted to contain semi-degraded repeats and ‘dark matter’ are similar in size to the entire *A. thaliana* nucleus (see also Figure 2A).

Our interpretations of the distribution of histone marks proposed above and summarized in Figure 3 are best confirmed with ChIP-seq, where we expect (i) genes and associated regulatory regions would be isolated with antibodies against H3K4 methylation, (ii) sequences comprising ‘dark matter’ (i.e., low/single copy, non-genic sequences) would be preferentially isolated with H3K9me3 and H3K27me3 antibodies, (iii) repetitive sequences would be isolated with H3K9me1 and H3K27me1 antibodies, potentially in proportions similar to the repeat profile across the genome as a whole (Table 1), and (iv) semi-degraded but still recognisable repeats in quantities intermediate between those observed in categories (ii) and (iii) would be isolated using antibodies against H3K27me2 and H3K9me2. It is also possible that there are distinctive repeats confined to this category of semi-degraded repeats.

### 3.2. The Genomic Organization of Plant Genomes Revealed by Histone Marks

Our interpretations, which are summarized in Figure 2C, predict that there are no fundamental differences in chromatin organization between the small genome of *A. thaliana* and the giant genome of *F. imperialis*, with the exception of the enormous differences in the total amount of repeats, semi-degraded repeats, and ‘dark matter’. In *A. thaliana*, repeats and semi-degraded repeats are co-localised in chromocentres and spatially separate from the genomic domains comprising genic regions and ‘dark matter’. While a model of an intermixing of repeats with semi-degraded repeats might also have been expected for *F. imperialis*, our interpretation of the results suggest that these domains are separate (Figure 3). One possible explanation is that in the repeat domains (i.e., light blue areas, Figure 3) and over very long tracks of the genome, repeat expansion and recombination-based processes cease to occur (or occur at a very low frequency), resulting in the repeats in this domain degrading and becoming long tracks of semi-degraded repeats and ‘dark matter’ (i.e., purple ‘spots’).

The different distribution of repeats from semi-degraded repeats and ‘dark matter’ in *F. imperialis* could be driven by the way chromosomes are organised and packaged in the 3D interphase nucleus. In species with small genomes, chromosomes typically occur in discrete territories [25], with particular chromatin types, e.g., constitutive and facultative heterochromatin forming distinctive domains spanning up to 100 kb [26]. In *F. imperialis*, these domains may span huge physical distances, possibly amounting to hundreds of Mbp, distances that could generate the more highly partitioned nuclear structure observed here.

After mitotic anaphase, chromosomes enter interphase in a conformation that reflects their anaphase mobility, the so-called Rabl [27] configuration with centromeres at one pole of the nucleus and telomeres at the other. Whilst a relic of this configuration may be visible in *A. thaliana* [26], small changes in DNA conformation have the potential to bring any bit of DNA into close proximity with any other [25] and to change the orientation of chromosomes. This has evolutionary consequences, as it provides opportunities for genome-wide DNA–DNA interactions, impacting sequence recombination, repeat mobility, and repair pathways, and hence, genome divergence. In species with larger genomes, the Rabl configuration is much more apparent [28], and we might predict that DNA–DNA interactions will be fundamentally different. The immense physical and spatial distances separating the DNA likely result in inter- and intra-chromosomal interactions being more highly constrained. If so, then local domains of sequence type, e.g., blocks of repeats or ‘dark matter’ will be freer to diverge independently of each other.

Recently Mei et al. [29] proposed that larger genomes have a more open chromatin configuration, i.e., chromatin that they call ‘functional space’, which can accumulate high levels of genetic diversity upon which selection acts across multiple independent loci, leading to “soft sweeps”. In contrast, plants with smaller genomes have less functional space, more local genetic variation, and are more prone to hard sweeps. Potentially, the antibodies against H3K9me3 and H3K27me3, which we propose as marks for ‘dark matter’, may represent the same domain as “functional space” reported by Mei et al. [29]. If so, then we can expect that these antibodies will label non-genic DNA sequences that are important in selection.

## 4. Materials and Methods

### 4.1. RepeatExplorer2 Analysis of Repeat Content

A total of 10.5 million (10,463,937) paired-end 100-bp Illumina HiSeq reads from genome skimming of *Fritillaria imperialis* leaf material (NCBI SRA accession number ERR845263) was used to estimate the repeat content of the genome using the pipeline ‘RepeatExplorer2’ [20,30]. These repeats represent approximately 2.3% of the genome, based on the GS of *F. imperialis* (45,000 Mbp/1C; [9]). Briefly, RepeatExplorer2 groups the Illumina reads into repeat clusters based on graph-based clustering of sequence reads, whereby each sequence read represents a node and edges between nodes represent similarity. All-to-all blast is performed on the sequence reads, with similarity hits (edge weights) registered where there is ≥90% sequence identity over ≥55% of the read length. Clusters are defined based on a maximum modularity approach to analysing the graph, and resulting clusters are grouped into superclusters based on paired-end sequence information (i.e., physical proximity in the genome). Each sequence cluster represents a distinct group of related repeats [20], and those clusters that contain ≥0.01% of input reads are used to estimate the proportion and identity of repeats present in the *F. imperialis* genome.

### 4.2. Immunolabelling

2C nuclei of *F. imperialis* were isolated, sorted, and immunolabelled, as described in Lysak et al. [31], with minor modifications. Briefly, approximately 10 mg of leaf tissue was fixed in ice-cold 4% (*w*/*v*) formaldehyde solution in 1 × phosphate-buffered saline (PBS) for 40 min. After washing with ice-cold Tris buffer for 2 × 10 min, the leaf tissue was chopped with a razor blade in 400 μL LB01 buffer [32] in a Petri dish on ice and filtered through a nylon mesh. Afterwards, 10 µL of sucrose buffer was mixed with 10 µL of the sorted 2C nuclei suspension and air-dried to a glass slide. Cells were then re-fixed with 4% (*w*/*v*) formaldehyde in 1 × PBS at room temperature for 30 min. After rinsing the slides with 1 × PBS for 2 × 5 min, the slides were incubated in 1% (*w*/*v*) bovine serum albumin (BSA) in 1 × PBS under parafilm in a moist chamber at 37 °C for 1 h. After rinsing the slides in 1 × PBS for 5 min, the slides were incubated with the appropriate antibodies (see Table 2) in 1 × PBS at 4 °C overnight in a moist chamber.

Indirect immunocytochemistry approaches were applied, using primary rabbit antibodies (Upstate^®^—now marketed by Merck), as described in Marques et al. [33] (see Table 2). After detection with anti-rabbit-rhodamine (Jackson ImmunoResearch Europe, Suffolk, UK), the slides were embedded in antifade (Vector Laboratories, Burlingame, CA, USA), supplemented with 0.5 mg/mL DAPI (Invitrogen, Carlsbad, CA, USA), and analysed with an Axioplan 2 epifluorescence microscope.

## Figures and Tables

**Table 1:** Summary of the abundance of repeats in the *Fritillaria imperialis* genome determined from analysing genome skimming of 2.3% of the genome using RepeatExplorer2.

Repeat Type	Genome Proportion
	(% of the Genome)
*LTR retrotransposons*	
Ty1/Copia-like	4.72
Ty3/Gypsy-like	18.91
LTR (other)	0.90
*Non-LTR retrotransposons*	
LINEs	0.19
*DNA transposons*	
EnSpm/CACTA-like	2.31
*Other*	
Pararetrovirus	0.38
Ribosomal DNA	0.22
Satellite repeats	0.25
Unknown	5.50
**TOTAL**	**33.4**

**Table 2:** List of antibodies targeting the different histone marks.

Histone Mark Detected	Antibody Used	Dilution Used	Catalogue Number in Upstate^®^
H3K4me1	Rabbit anti-H3K4me1	1:200	07-436
H3K4me2	Rabbit anti-H3K4me2	1:300	07-030
H3K9me1	Rabbit anti-H3K9me1	1:200	07-395
H3K9me2	Rabbit anti-H3K9me2	1:300	07-441
H3K9me3	Rabbit anti-H3K9me3	1:300	07-473
H3K27me1	Rabbit anti-H3K27me1	1:100	07-448
H3K27me2	Rabbit anti-H3K27me2	1:50	07-452
H3K27me3	Rabbit anti-H3K27me3	1:100	07-449

## Data Availability

Immunocytochemical labelling data are contained within the article and in Fuchs and Schubert [21]. Illumina sequences used for repeat analysis with RepeatExplorer2 are available via NCBI (SRA accession number ERR845263).
